# Fibroblast growth factor 21 is expressed and secreted from skeletal muscle following electrical stimulation *via* extracellular ATP activation of the PI3K/Akt/mTOR signaling pathway

**DOI:** 10.3389/fendo.2023.1059020

**Published:** 2023-02-23

**Authors:** Manuel Arias-Calderón, Mariana Casas, Julián Balanta-Melo, Camilo Morales-Jiménez, Nadia Hernández, Paola Llanos, Enrique Jaimovich, Sonja Buvinic

**Affiliations:** ^1^ Institute for Research in Dental Sciences, Faculty of Dentistry, Universidad de Chile, Santiago, Chile; ^2^ Institute of Biomedical Sciences, Faculty of Medicine, Universidad de Chile, Santiago, Chile; ^3^ Faculty of Medicine, Center for Exercise, Metabolism and Cancer Studies CEMC, Universidad de Chile, Santiago, Chile; ^4^ School of Dentistry, Faculty of Health, Universidad del Valle, Cali, Colombia; ^5^ Department of Basic Sciences of Health, Faculty of Health Sciences, Pontificia Universidad Javeriana, Cali, Colombia

**Keywords:** fibroblast growth factor 21, extracellular nucleotides, myokines, Akt signaling, excitation-transcription coupling, membrane depolarization, muscle activity

## Abstract

Fibroblast growth factor 21 (FGF21) is a hormone involved in the regulation of lipid, glucose, and energy metabolism. Although it is released mainly from the liver, in recent years it has been shown that it is a “myokine”, synthesized in skeletal muscles after exercise and stress conditions through an Akt-dependent pathway and secreted for mediating autocrine and endocrine roles. To date, the molecular mechanism for the pathophysiological regulation of FGF21 production in skeletal muscle is not totally understood. We have previously demonstrated that muscle membrane depolarization controls gene expression through extracellular ATP (eATP) signaling, by a mechanism defined as “Excitation-Transcription coupling”. eATP signaling regulates the expression and secretion of interleukin 6, a well-defined myokine, and activates the Akt/mTOR signaling pathway. This work aimed to study the effect of electrical stimulation in the regulation of both production and secretion of skeletal muscle FGF21, through eATP signaling and PI3K/Akt pathway. Our results show that electrical stimulation increases both mRNA and protein (intracellular and secreted) levels of FGF21, dependent on an extracellular ATP signaling mechanism in skeletal muscle. Using pharmacological inhibitors, we demonstrated that FGF21 production and secretion from muscle requires the activation of the P2YR/PI3K/Akt/mTOR signaling pathway. These results confirm skeletal muscle as a source of FGF21 in physiological conditions and unveil a new molecular mechanism for regulating FGF21 production in this tissue. Our results will allow to identify new molecular targets to understand the regulation of FGF21 both in physiological and pathological conditions, such as exercise, aging, insulin resistance, and Duchenne muscular dystrophy, all characterized by an alteration in both FGF21 levels and ATP signaling components. These data reinforce that eATP signaling is a relevant mechanism for myokine expression in skeletal muscle.

## 1 Introduction

Fibroblast growth factor 21 (FGF21) is a pleiotropic peptide hormone. The physiology of FGF21 is largely complex because it is synthesized and secreted by several organs and can act on multiple target tissues in either a paracrine or an endocrine fashion (1, 2). First described expressed in liver cells (2), it is now well considered an adipokine (3), myokine (4), and cardiomyokine (5). Furthermore, the molecular mechanism of FGF21 signaling is complex and involves several FGF receptors (FGFRs) as well as an obligate coreceptor, β-klotho (KLB). Tissue specificity of FGF21 signaling is conferred by the co-expression of a given FGF receptor and KLB (6). Some of the main physiological effects of FGF21 described to date are to increase the incorporation of glucose into cells, increase sensitivity to insulin, promote the use of fats in metabolism, decrease body mass index and glycemia and decrease insulin levels. It is a starvation-like hormone with metabolic functions that lead to maintaining fuel support to tissues (1, 7, 8).

In skeletal muscle, FGF21 is poorly expressed at rest (9, 10). However, different physiological and pathological conditions promote the expression and secretion of FGF21 in muscle. Muscle-derived FGF21 increases with starvation, endoplasmic reticulum stress, and mitochondrial dysfunctions (4, 11–14), as a response and adaptation factor to cellular stress mechanisms (15, 16). It has been proposed as a biomarker of muscle-specific mitochondrial disorders (17, 18). In particular, it has been described that events that alter mitochondrial function and increase oxidative stress levels could be the stimuli that induce FGF21 expression in skeletal muscle (11, 19, 20). Insulin has also been described as a strong stimulus for FGF21 expression in skeletal muscle. Both insulin infusion in healthy young men and pathological hyperinsulinemic condition renders to an elevated FGF21 expression in skeletal muscle (9). It has been described that exercise is also an important stimulus for the regulation of FGF21 expression in skeletal muscle. Aerobic exercise has been strongly associated with the increase in FGF21 plasma levels in humans (as reviewed in (21, 22)); it has been suggested derived from the liver, but tissue source of plasmatic FGF21 has not been addressed in those reports (23–28). Emerging evidence suggests that FGF21 from skeletal muscle could also contribute to increased plasma levels after exercise (27). The fact that FGF21 levels also increase in response to exercise fits to myokines definition: they are produced and secreted in response to muscle contractile activity (29), dependent on the depolarization of the sarcolemma. The expression of FGF21 in muscle in response to stress signals and during exercise makes sense. It has been described that cellular stress responses are associated with the molecular mechanisms of exercise and that exercise could induce its beneficial effects and mediate adaptation mechanisms in skeletal muscle through controlled stress signals, also regulating the expression of myokines (15, 16, 30–33).

A paradoxical situation, that illustrates the complexity of the FGF21 pathways, is that while its plasma levels increase during exercise, they also do so in processes of metabolic deregulation, such as obesity or liver diseases (22). In these latter cases, exercise even reduces plasma and liver levels of FGF21, and overexpresses its signaling pathway in the liver (34). In this way, it is now suggested that both the stimuli and the local/systemic responses depend on the source organ that produces FGF21. In this way, an increase in “metabolic” plasma FGF21 (released from the liver or adipocytes) would not be equivalent to “exercise” FGF21 (released by skeletal muscles) (22). Exercise increases FGF21 levels associated with protein kinase B/Akt1 protein activation (15, 24, 35). FGF21 expression and secretion is improved in C2C12 muscle cells transduced with a constitutively active form of Akt1 (4). In addition, muscle-specific Akt1 transgenic mice increase both mRNA and protein level of FGF21 in muscles, as well as FGF21 serum levels (4). An increase in muscle FGF21 has been also described in a transgenic animal model with constitutively-activated mammalian target protein of Rapamycin (mTOR) (36). These data suggest that FGF21 is under the control of the PI3K-Akt-mTORC1 signaling pathway in skeletal muscle.

Direct effects of FGF21 on muscle cells have been also described, suggesting a putative autocrine/paracrine role of this myokine. It has been described that FGF21 induces glucose uptake in skeletal muscle, through a mechanism that does not involve the canonic Akt-dependent glucose uptake pathway (37). We recently published that FGF21 regulates glucose uptake in adult skeletal muscle fibers through a mechanism dependent on both GLUT4 translocation to cell surface and atypical PKC-ζ activation (38). On the other hand, Zhou et al. have described that the expression of FGF21 is increased in muscles of mdx mice, a model of Duchenne muscular dystrophy (39, 40). Also, it has been described that FGF21 could mediate muscle plasticity processes by inducing an increase in aerobic fibers, both *in vitro* and *in vivo* (41). Moreover, FGF21 is responsible for muscle atrophy associated with metabolic alterations (12, 42). To date, the exercise-cellular stress relationship appears to be the main axis for the induction of FGF21 in skeletal muscle, and that the production of this myokine would be associated with the activation of the Akt pathway. However, the molecular mechanism that regulates the expression and secretion of this factor from normal skeletal muscle is still unknown, as is the initial stimulus that would allow the activation of the pathway associated with the Akt protein for the regulation of muscle FGF21. Classically, the activation of Akt is associated with the activation of the PI3K protein through stimulation of receptor tyrosine kinases or G protein-coupled receptors (43). Downstream of Akt is the protein mTORC1 which has also been associated with controlling FGF21 in skeletal muscle (36). This signaling pathway is activated by exercise, an important muscle FGF21-inducing stimulus (24). Therefore, it is interesting to study a mechanism that relates the activation of this pathway in normal muscle conditions, unlike most of the published data that use an altered expression of FGF21 by genetic tools or in pathological conditions.

We have previously described that exercise adaptation mechanisms in skeletal muscle are regulated by extracellular ATP (eATP)-mediated signaling, which activates the Excitation-Transcription (ET) coupling (44–47). eATP is released from muscle cells after membrane depolarization and activates their P2X/P2Y purinergic receptors to evoke cytosolic Ca^2+^ transients related to gene expression (45, 47). This mechanism is also related to the synthesis and release of interleukin 6 (IL6), a well-known myokine, in response to electrical stimulation (48, 49). Consequently, the ET-coupling, through signaling mediated by eATP, is a possible pathway to study as a molecular mechanism that associates exercise with the production of myokines in skeletal muscle. It has been described that ET-coupling activates Akt in muscle fibers in response to electrical stimulation, through the PI3K/Akt pathway (50). Moreover, we have recently demonstrated that eATP induces protein synthesis in whole *flexor digitorum brevis* (FDB) muscle *in vitro* through the P2Y/PI3K/Akt/mTOR signaling pathway (51). In addition, eATP signaling has been shown to play a role in cellular stress-dependent adaptation, *via* reactive oxygen species (52). Recent evidence from our laboratory indicates that ET-coupling is related to mitochondrial stress events in response to electrical stimulation, *via* IP3-dependent Ca^+2^ signals (53). Both Akt activation and cellular stress signals are related to FGF21 production in skeletal muscle, so it is interesting to study the role of eATP in the control of FGF21 expression and secretion in skeletal muscle. The relationship of the signaling *via* eATP with the regulation of FGF21 is also supported by reports of direct effects of FGF21 on skeletal muscle (glucose uptake (37), formation of aerobic fibers (41), control of muscle mass (12) and its alterations in pathological conditions such as Duchenne muscular dystrophy (40)), situations in which our laboratory has reported that signaling mediated by eATP participates directly, or is altered (47, 50, 54).

Considering this background, it is necessary to elucidate the molecular mechanism that controls the expression and secretion of FGF21 in skeletal muscle. We here show that electrical stimulation elicits FGF21 synthesis and secretion in skeletal muscle, by eATP activation of a P2YR/PI3K/Akt/mTORC1 pathway. These results allow us to identify the extracellular ATP-dependent signaling pathway as a new target to modulate the production of FGF21 in skeletal muscle, as well as to incorporate FGF21 as one of the genes regulated by ET coupling.

## 2 Materials and methods

All procedures involving animals were approved by the Institutional Animal Care and Use Committee of the Faculty of Dentistry of Universidad de Chile (Certificate N° 061501). The results are reported following the ARRIVE guidelines.

### 2.1 Muscle dissection and stimulation

Male BALB/c mice (8 weeks old, 18-25 g) were obtained from the Experimental Platform of the Faculty of Dentistry (Universidad de Chile). Standard animal room conditions (48–50% humidity; 20 ± 2°C; 12 h light/dark cycle), and *ad libitum* water and food (LabDiet^®^ JL Rat and Mouse/Auto 6F 5K67) were maintained. FDB muscles were isolated from BALB/c mice as previously described (46), and stabilized for 2 h in DMEM (Thermo Fisher Scientific, MA, USA) supplemented with 1 mM sodium pyruvate (Sigma-Aldrich Corp, St. Louis, MO, USA), 100 U/mL penicillin (Thermo Fisher Scientific, MA, USA), 100 µg/mL streptomycin (Thermo Fisher Scientific, MA, USA) and 1% horse serum (Thermo Fisher Scientific, MA, USA), at 37° C.

### 2.2 Isolation of adult skeletal fibers

Isolated fibers from the FDB muscle were obtained by enzymatic digestion with collagenase type II (90 min with 400 U ml^−1^) and mechanic dissociation with fire-polished Pasteur pipettes, as previously described (46). The isolated fibers were seeded in ECM-coated dishes and used 20 h after seeding.

### 2.3 Electrostimulation *in vitro* or *in situ*

Isolated FDB muscle fibers were electrically stimulated *in vitro* following the protocol previously established in our laboratory (47). A field electrode, covering the entire surface of the plate on which the isolated fibers are cultured, connected to a Grass S48 pulse generator was used. The stimulation was performed at 20 Hz (270 pulses, 0.3 ms each; 2 mV), a frequency that induces the maximum release of ATP from the muscle fibers (47). For *in-situ* stimulation, the same equipment and stimulation pattern described were used. In this experimental condition, male BALB/c mice (6-8 weeks) were anesthetized by intraperitoneal injection of 80 mg/kg ketamine and 8 mg/kg xylazine. Subsequently, an incision was made at the level of the lower extremities, in the upper lateral part of the gastrocnemius muscle, to directly stimulate the sciatic nerve (20 Hz, 270 pulses, 0.3 ms each; 0.3 mV). After stimulation, the animal was euthanized by cervical dislocation, the FDB muscles were dissected and kept in DMEM medium (Thermo Fisher Scientific, MA, USA) supplemented with 10% fetal bovine serum (Biological Industries; CT, USA), at 37°C for 2 h before processing for protein analysis. In this model, an FDB muscle was considered as the experimental condition, and the contralateral muscle was used as intra-animal control, a muscle that underwent the same surgical procedure, but without the application of electrical stimulation.

### 2.4 ATP concentration- and time-response curves. Effect of antagonists and blockers

To determine the effect of ATP (Adenosine 5′-triphosphate, Sigma-Aldrich Corp, St. Louis, MO, USA) on either FGF21 protein or mRNA levels, isolated fiber cultures or FDB muscles were previously serum-starved for 2 h (DMEM culture medium without horse serum). Subsequently, muscles were stimulated with exogenous ATP at selected concentrations (0.1-100 µM), for different times (30-360 min). When blockers or inhibitors were used, they were incubated 30 min before and during the stimulation with ATP. Evaluation of changes in mRNA and protein levels in response to ATP was performed in the presence of 100 μM Suramin (Sigma-Aldrich Corp, St. Louis, MO, USA), 25 μM Nifedipine (Sigma-Aldrich Corp, St. Louis, MO, USA), 100 nM Rapamycin (Sigma-Aldrich Corp, St. Louis, MO, USA), 50 μM LY294002 (Cell Signaling Technology, Danvers, MA, EEUU), 10 μM Akt VIII (Sigma-Aldrich Corp, St. Louis, MO, USA) 30 μM Cycloheximide (Sigma-Aldrich Corp, St. Louis, MO, USA) or 0.5 μM actinomycin-D (Sigma-Aldrich Corp, St. Louis, MO, USA).

### 2.5 Total RNA extraction, reverse transcription and quantitative real-time PCR

Total mRNA was obtained from cell cultures using Trizol™ reagent (Life Technologies, CA, USA), according to the manufacturer’s instructions. cDNA was obtained from 2 µg of total RNA by using the High-Capacity cDNA Reverse Transcription Kit (#4368814, Applied Biosystems, CA, USA), as indicated by the manufacturer´s protocol.

The qRT-PCR was carried out in the StepOne™ Real-Time PCR System (Thermo Fisher Scientific, Waltham, MA, USA) using the Brilliant III Ultra-Fast SYBR^®^ Green QPCR Master Mix (#600882, Agilent Technologies, CA, USA). The sequences of the primers used to amplify the cDNA were: FGF21 (600 nM) sense: TACACAGATGACGACCAAGA; antisense: GGCTTCAGACTGGTACACAT; and Glyceraldehyde-3-phosphate dehydrogenase (GAPDH) (400 nM) sense: CAACTTTGGCATTGTGGAAG, antisense: CTGCTTCACCACCTTCTTG. All primers were standardized to render an efficiency between 95% and 105%. The thermocycling protocol included 95 °C for 3 min followed by 40 cycles of 95 °C for 20 s and 60 °C for 20 s. The amplification procedure was verified by melting curve analysis. The results were normalized to GAPDH expression (housekeeping) and reported according to the 2^-ΔΔCT^ method (55).

### 2.6 Quantitative measurement of secreted FGF21

To determine the concentration of FGF21 secreted into the culture medium from FDB muscle stimulated with ATP, the commercial Mouse FGF21 ELISA kit ab212160 (Abcam, Cambridge, U.K) was used following the instructions provided by the manufacturer.

### 2.7 Immunoblot

FDB muscles were processed with a rotor/stator tissue homogenizer (Biospec, OK, USA) in 150 µl of ice-cold lysis buffer (20 mM Tris-HCl, 1% Triton X-100, 2 mM EDTA, 10 mM Na_3_VO_4_, 20 mM NaF, 10 mM sodium pyrophosphate, 150 mM NaCl, 1 mM PMSF, 1:200 protease inhibitor cocktail Calbiochem Set III, pH 7.4). The cell lysates were sonicated for 3 min, incubated on ice for 30 min, and centrifuged to remove debris. The protein concentration was determined by the turbidimetric assay with sulfosalicylic acid. Proteins resolution by 10% SDS-PAGE and immunoblot were performed as previously detailed (48). Protein staining was performed with the RapidStepTM enhanced chemiluminescence (ECL) reagent (EDM Millipore, MA, USA). Images were acquired in an Amersham Imager 600 (GE Healthcare Life Sciences, PA, USA) and densitometry was analyzed with the ImageJ Software (NIH, MA, USA). Monoclonal antibodies were used for detection of FGF21 (0.4 μg/ml, # ab171941, Abcam, Cambridge, UK) or the loading control GAPDH (1 μg/ml, #G9545, Sigma-Aldrich Corp, St. Louis, MO, USA).

### 2.8 Statistical analysis

Data of n experiments were expressed as mean ± standard error of the mean (SEM). Non-parametric tests were used to evaluate significance. Mann-Whitney test was used for comparing a single condition with a control. For multiple comparisons, the Kruskal Wallis test followed by the Dunn *post hoc* test was used. A p value < 0.05 was considered statistically significant. Statistical analyzes were performed using the Graph Pad Prism 6 software (CA, USA).

## 3 Results

### 3.1 Electrical stimulation increases FGF21 mRNA and protein levels, through eATP signaling

**Figure 1:**
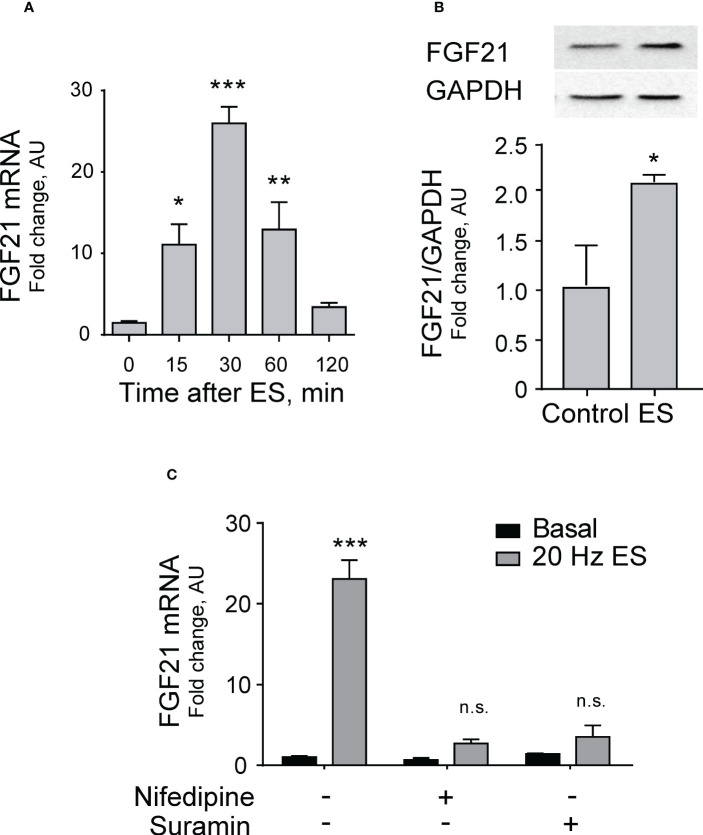
Electrical stimulation increases FGF21 expression dependent on the eATP signaling pathway in skeletal muscle. **(A)** Electrical stimulation (ES, 20 Hz, 270 pulses, 0.3 ms each) evokes a transient increase in FGF21 mRNA levels in FDB isolated muscle fibers. n=6; *p<0.05; ***p<0.001 vs Control; Kruskal-Wallis with Dunn’s post-hoc test. **(B)** *In situ* electrical stimulation (20 Hz, 270 pulses, 0.3 ms each) of sciatic nerve evokes an increase in FGF21 protein levels in whole-FDB muscle, 120 min after stimulation. n=4; *p<0.05 vs Control; Mann-Whitney test. **(C)** Nifedipine (25 μM), a blocker of Cav1.1-pannexin-1 communication, and suramin (100 μM), a non-selective P2Y/P2X receptors antagonist, both reduce the increase in FGF21 mRNA levels evoked by electrical stimulation (20 Hz, 270 pulses, 0.3 ms each), 30 min after stimulation, in FDB isolated muscle fibers. n=4; n.s., not significant; ***p<0.001 vs Basal; Kruskal-Wallis with Dunn’s post-hoc test.

Electrical stimulation (20 Hz, 270 pulses, 0.3 ms each) of FDB isolated muscle fibers evoked a significant increase in FGF21 mRNA levels measured at different times after stimulation; the peak was reached at 30 min with more than 25-fold increase (**Figure 1A**).

To analyze changes in protein expression of FGF21 after membrane depolarization, an *in situ* electrical stimulation of the sciatic nerve was performed in mice, which corresponds to the neural branch that innervates the entire hindlimb, including the FDB. A two-fold increase in FGF21 protein level was observed in FDB whole muscle 120 min after electrical stimulation (**Figure 1B**).

We tested the hypothesis that FGF21 expression could be mediated by the pathway that links electrical stimulation to ATP release through pannexin-1 channels activated by the voltage sensor Cav1.1 to stimulate P2Y purinergic receptors. To that aim, we studied the increase in FGF21 mRNA levels after electrical stimulation in the presence of drugs that block either the Cav1.1-pannexin-1 communication (nifedipine) or the P2Y purinergic receptors (Suramin). Both drugs reduced FGF21 mRNA expression to levels not significantly different from basal (**Figure 1C**), suggesting that indeed the effect of electrical stimulation is mediated by the ATP release signaling process.

**Figure 2:**
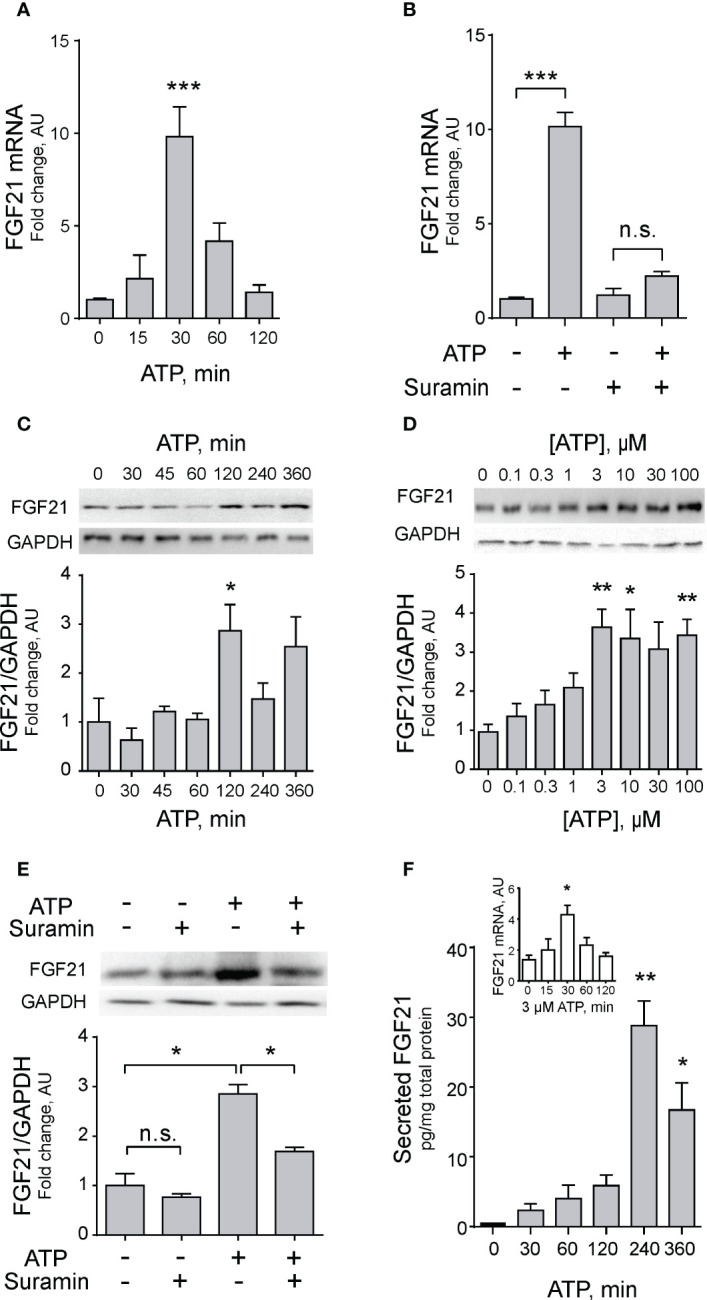
Exogenous ATP promotes FGF21 expression and secretion in skeletal muscle. **(A)** Exogenous ATP (100 μM) evokes a transient increase in FGF21 mRNA levels in FDB isolated muscle fibers. n=6; ***p<0.001 vs Control; Kruskal-Wallis with Dunn’s post-hoc test. **(B)** Suramin (100 μM), a non-selective P2Y/P2X receptors antagonist, decreases the effect of exogenous ATP (100 μM) on FGF21 mRNA levels, 30 min after stimulation, in FDB isolated muscle fibers. n=4; n.s., not significant; ***p<0.001; Kruskal-Wallis with Dunn’s post-hoc test. **(C)** Exogenous 100 μM ATP increases FGF-21 protein level in whole-FDB muscle extracts, at 120 min. n=3; *p<0.05 vs 0; Kruskal-Wallis with Dunn’s post-hoc test. **(D)** Exogenous ATP stimulation increases FGF-21 protein levels from 3 μM in whole-FDB muscle. n=4; n.s., not significant; *p<0.05, **p<0.01 vs 0; Kruskal-Wallis with Dunn’s post-hoc test. **(E)** Suramin (100 μM), a non-selective P2Y/P2X receptors antagonist, decreases the effect of exogenous 100 μM ATP on FGF21 protein levels, at 120 min of stimulation, in whole-FDB muscle extracts. n=4; n.s., not significant; *p<0.05; Kruskal-Wallis with Dunnett’s post-hoc test. **(F)** Exogenous 3 μM ATP stimulation increases FGF21 secretion to extracellular medium from whole-FDB muscle, at 240 min. n=4; *p<0.05, **p<0.01 vs Control; Kruskal-Wallis with Dunn’s post-hoc test. The inset shows that 3 μM ATP concentration also increases mRNA levels of FGF21 in skeletal muscle fibers, as previously demonstrated with 100 μM eATP **(C)**.

To further explore this mechanism, we incubated muscle fibers at different times in the presence of 100 µM eATP. The increase in FGF21 mRNA peaked at 30 min with a 10-fold increase (**Figure 2A**). Confirming the role of the purinergic signaling, in fibers incubated with 100 μM suramin this increase was abolished (**Figure 2B**). Accordingly, FGF21 protein expression increased in FDB muscles after 120-min incubation with 100 µM ATP (**Figure 2C**). A dose-response curve with different ATP concentrations showed that maximal protein expression occurs at 3 µM extracellular ATP with little or no reduction at higher concentrations (**Figure 2D**). Of note, the increase in protein expression was also inhibited by incubation with 100 µM suramin (**Figure 2E**).

An important question about newly produced FGF21 is whether it is stored/degraded or secreted from muscle fibers. The secretion of FGF21 was addressed by ELISA assays in the extracellular media of muscle fibers incubated with 3 µM extracellular ATP. This reduced ATP concentration was used considering that was the smallest concentration that evoked significant increases in FGF21 protein expression (**Figure 2D**), and that this concentration has been reported to selectively activates P2Y but not P2X purinergic receptors (56, 57). A significant 30-fold increase in secreted FGF21 was observed at 240 min incubation with exogenous ATP (**Figure 2F**). As a validation of previous results with 100 µM ATP, it was demonstrated that the 3 µM concentration also increases FGF21 mRNA expression after 30-min incubation (**Figure 2F**, inset).

### 3.2 FGF21 expression and secretion is regulated by transcriptional activation *via* the PI3K-Akt-mTOR pathway

**Figure 3:**
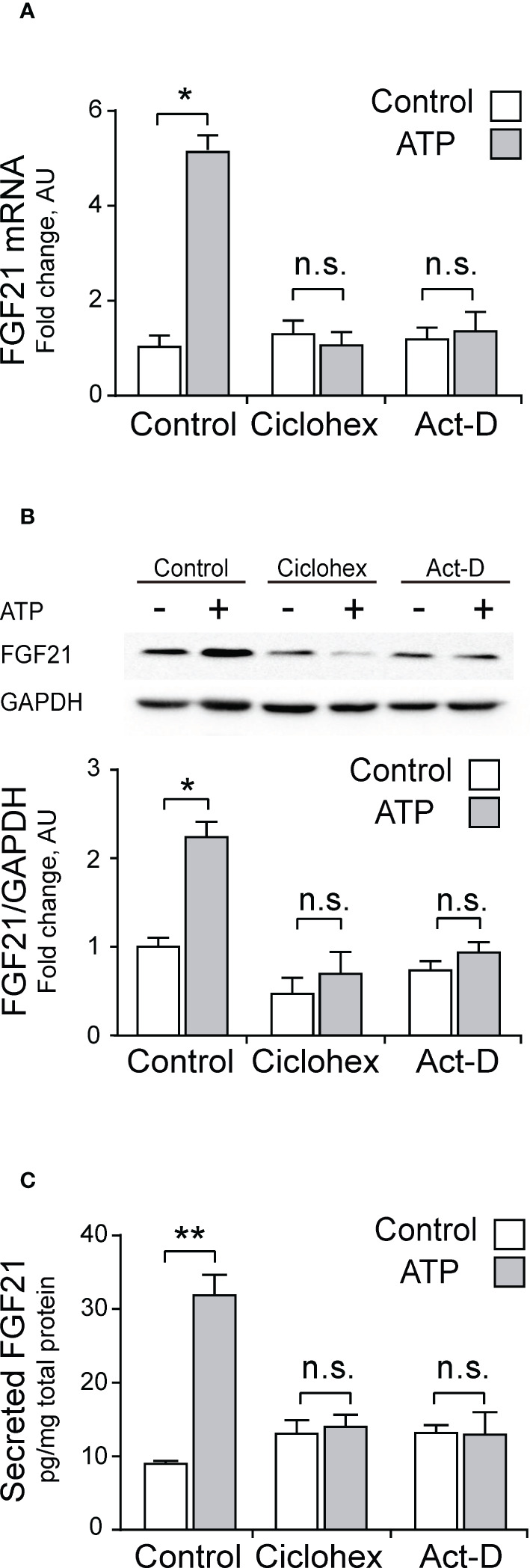
ATP stimulation increases FGF21 secretion, protein content, and mRNA levels in whole-FDB muscle, through a transcription-mediated mechanism. Cycloheximide (30 μM; Ciclohex), a general translation inhibitor, and Actinomycin-D (0.5 μM; Act-D), a general transcription inhibitor, both abolished the effect of 3 μM ATP stimulation over mRNA **(A)**, protein **(B)**, and secreted **(C)** FGF21 levels. n=4; n.s., not significant; *p<0.05, **p<0.01 vs non-ATP Control; Mann-Whitney test.

To make sure that mRNA expression, protein synthesis and secretion of FGF21 are indeed mediated by the transcriptional machinery of the skeletal muscle fiber, we studied the effect of 3 µM exogenous ATP in the whole FDB muscle in the presence of either 30 µM cycloheximide (a general inhibitor of translation) or 0.5 µM actinomycin-D (a general inhibitor of transcription). As shown in **Figure 3**, both inhibitors completely abolished the effect of ATP on FGF21 mRNA levels (**Figure 3A**), FGF21 protein levels (**Figure 3B**) and FGF21 secreted levels (**Figure 3C**), indicating that this process is regulated by transcription.

**Figure 4:**
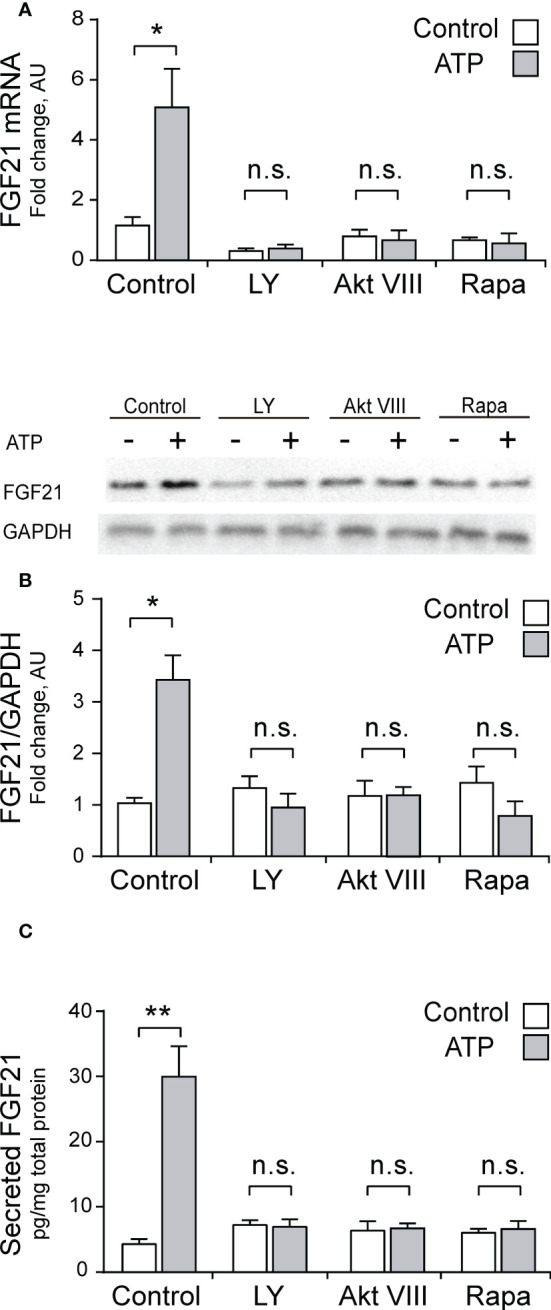
Pharmacological inhibition of PI3K-Akt-mTOR pathway abolished the increase in mRNA, protein and secreted FGF21 levels evoked by ATP stimulation, in whole-FDB muscle. LY294002 (50 μM), a general PI3K inhibitor, Akt VIII (10 μM), an Akt inhibitor, and Rapamycin (100 nM), a mTORC1 inhibitor, all blocked the 3 μM ATP stimulation effect on mRNA **(A)**, protein **(B)**, and secreted **(C)** FGF21 levels, in whole-FDB muscle. n=4; n.s., not significant; *p<0.05, **p<0.01, vs non-ATP Control; Mann-Whitney test.

Considering than Akt has been described as a classical regulator of FGF21 (36, 43), and our previous reports showing that the eATP pathway in skeletal muscle activates the Akt signaling pathway (50, 51), we studied the PI3K-Akt-mTOR signaling pathway as a putative target downstream the P2Y receptors for regulation of FGF21 expression in skeletal muscle. LY294002 (50 μM), a general PI3K inhibitor, Akt VIII (10 μM), an Akt inhibitor, and Rapamycin (100 nM), a mTORC1 inhibitor, all blocked the stimulation effect of 3 μM ATP over mRNA, protein, and secreted FGF21 levels, in whole-FDB muscle (**Figures 4A–C**). This is strong evidence in favor of the involvement of PI3K-Akt-mTOR pathway downstream of purinergic stimuli in skeletal muscle fibers.

## 4 Discussion

**Figure 5:**
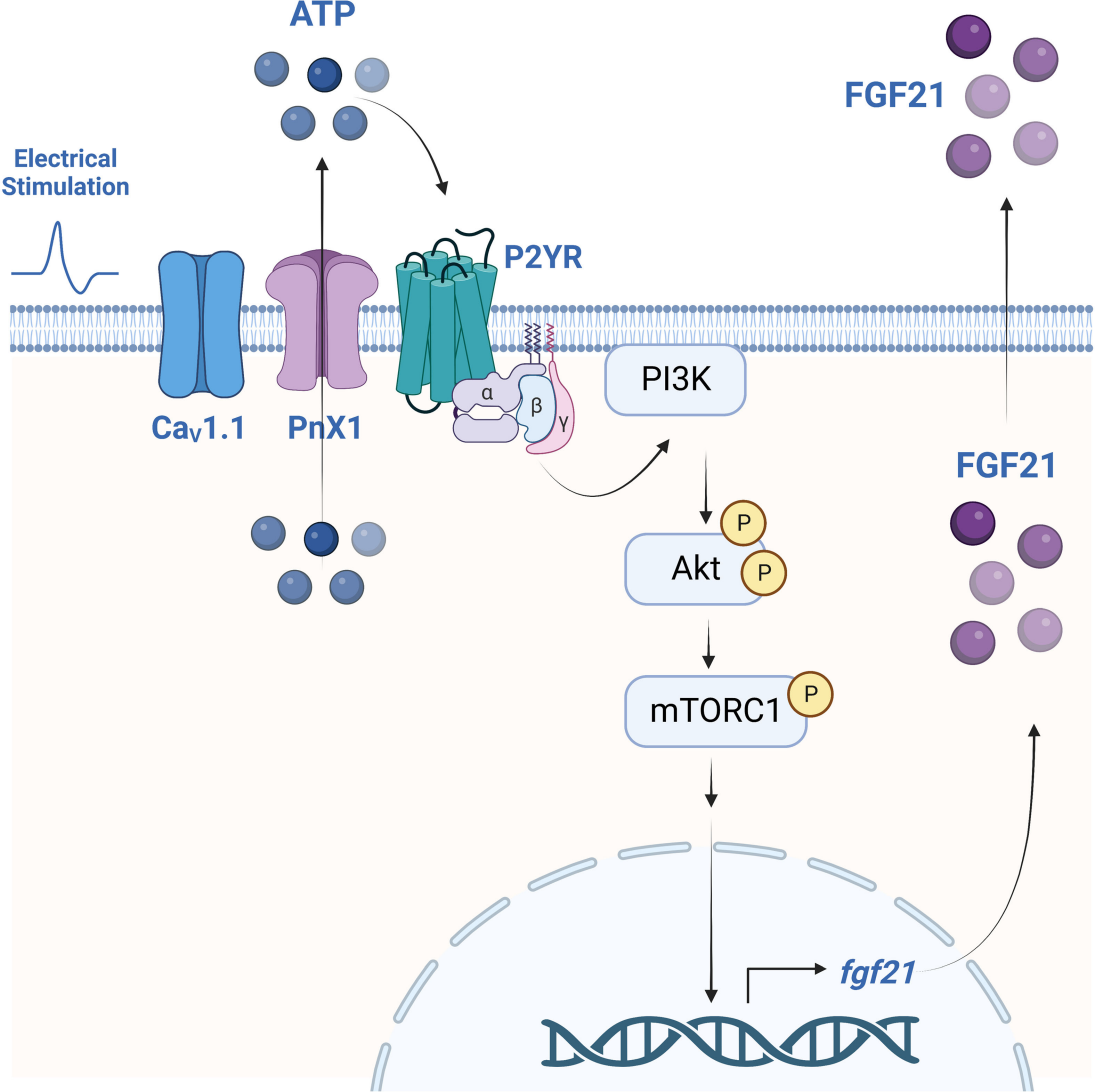
Proposed model for the regulation of FGF21 expression and secretion by electrical stimulation, dependent on extracellular ATP signaling and activation of the P2YR/PI3K/Akt/mTORC1 pathway in mouse skeletal muscle. Electrical stimulation is sensed by the dihydropyridine receptor (Ca_V_1.1) that, as we previously reported, evokes ATP release from muscle cells through Pannexin-1 hemichannels (PnX1). eATP stimulates P2Y receptors (P2YR) that activate the PI3K/Akt/mTORC1 signaling pathway to promote FGF21 expression and secretion. Created with BioRender.com.

In recent years, FGF21 has gained attention as an important regulator of metabolic processes at the systemic level, with multiple beneficial effects in different pathologies such as diabetes, insulin resistance, and obesity, among others (42, 58, 59). FGF21 is expressed in different tissues in humans and murine research models, one of them being skeletal muscle, in which it has been categorized as a myokine (29, 32, 42). However, the muscle regulation mechanisms of this factor have been mainly associated with pathologies or physiological alterations that induce the expression of FGF21 (42), so its production in muscle under physiological conditions is still controversial. One of the main physiological stimuli reported to regulate FGF21 in skeletal muscle is exercise (35, 58–60). However, the molecular mechanism by which exercise could induce the expression and secretion of FGF21 is unknown. We here demonstrate for the first time the role of electrical stimulation through extracellular ATP in the regulation of the expression, synthesis, and secretion of FGF21 by skeletal muscle through the activation of the P2YR/PI3K/Akt/mTORC1 pathway, as summarized in the graphic model of **Figure 5**. These results allow us to identify the extracellular ATP-dependent signaling pathway as a new study target to modulate the production of FGF21 in skeletal muscle and incorporate FGF21 as one of the genes associated with the control of ET coupling, acting as a paracrine regulator of muscle plasticity.

At the molecular level, exercise can be studied *in vitro* in muscle cell cultures through the application of electrical stimulation (61), thus allowing the study of molecular events related to exercise and its responses, such as the control of the production of myokines. In this context, it is interesting to study whether the electrical stimulus regulates the expression, synthesis, and secretion of FGF21 in skeletal muscle. Our laboratory has reported that the electrical stimulation induces the release of ATP into the extracellular space, acting as a relevant mediator of ET coupling (44, 45). This pathway controls the expression of different genes associated with muscle plasticity, including IL-6 as a myokine (48, 49). However, until now, a relationship between this pathway and the expression of FGF21 has not been studied. The electrical stimulation parameters that promote the highest ATP release through Pannexin1 from skeletal fibers in previous studies (20 Hz, 270 pulses, 0.3 ms each) (47) increased levels of FGF21 mRNA, intracellular protein, and secreted protein. The depolarization-evoked increase in FGF21 mRNA was abolished when P2Y/P2X receptors were antagonized by Suramin, reinforcing the role of eATP in this process. The effect of as low as 3 µM of exogenous ATP to promote FGF21 protein expression suggests that P2Y receptors are involved because they respond to nM-low µM ATP concentrations. In contrast, P2X requires high µM to mM ATP for activating (56, 57). Within the subtypes of P2Y receptors identified to date, it has been described that the P2Y_2_ receptor is strongly expressed in isolated FDB muscle fibers (49) and that it is also part of the protein complex involved in the ET coupling (44), which suggests that this receptor could be involved in the regulation of FGF21 dependent on extracellular ATP. However, experiments are required to demonstrate the participation of P2Y_2_R in this mechanism specifically. In addition, the depolarization-evoked increase in FGF21 mRNA was abolished after Nifedipine incubation, which we have reported disturbs the relation between the voltage sensor Cav1.1 and the ATP releaser conduit Pannexin 1 (62). Interestingly, we have demonstrated that Cav1.1, Pannexin 1, P2Y receptors, and signaling molecules such as PI3K are joined as a multiprotein complex in the T-tubule of the skeletal muscle (44).

The increased FGF21 mRNA in skeletal isolated fibers electrically stimulated was reinforced with a more physiological approach using *in situ* sciatic nerve stimulation. When electrical stimulation was applied to the sciatic nerve in anesthetized mice, there was an increase in protein levels of FGF21 in the FDB muscle. Therefore, in a model where the neuromuscular junction is physiologically working, the result is similar than observed in isolated skeletal muscle fibers directly stimulated.

The direct incubation of whole FDB muscles with 100 μM exogenous ATP *in vitro*, showed a time-dependent increase in FGF21 mRNA and intracellular protein, which was abolished by preincubation with Suramin. Hence, direct activation of P2Y/P2X receptors promotes FGF21 expression. Interestingly, the maximal value in mRNA level of FGF21 evoked by eATP was observed at 30 min, while intracellular protein level was at 120 min and secreted FGF21 level was at 240 min. The latter suggests that secretion is linked to the expression and synthesis of FGF21; therefore, secretion does not operate as an independent mechanism. The differential time course for the increase in mRNA-protein-secretion levels suggests that the secretion of FGF21 requires the *de novo* synthesis of this factor. The same has been described for the family of endocrine growth factors, a classification in which FGF21 is found, in which its synthesis leads to its rapid secretion, without maintaining intracellular storage (19, 63). That hypothesis was confirmed in the current work when muscle stimulation with exogenous ATP was addressed after incubation with Actinomycin D (transcription blocker) or Cycloheximide (translation blocker). Both treatments prevented the ATP-evoked increase in FGF21 mRNA, intracellular protein, and secretion.

It has been described that the regulation of FGF21expression is associated with different signaling proteins. Izumiya et al. observed increased levels of FGF21 (mRNA and protein) in a transgenic model for constitutively active Akt, muscle-specific, suggesting that FGF21 expression depends on Akt activity in skeletal muscle (4). The same authors describe the participation of PI3K in the expression of this myokine (4). Furthermore, Guridi and colleagues have suggested that mTORC1 is also an important regulator of muscle production of FGF21 (36). In the current work, we tested the hypothesis that extracellular ATP activates the Akt-dependent signaling pathway to induce the expression and secretion of FGF21. Osorio-Fuentealba et al. observed an increase in PI3K-dependent Akt phosphorylation (Thr308 and Ser473) in response to stimulation with extracellular ATP in a myotube model, demonstrating an Akt activation mediated by purinergic receptor signaling (50). In agreement, we have recently published that 3 μM ATP is sufficient to activate the PI3K/Akt/mTORC1 signaling pathway within 20 min of stimulation in mouse FDB muscle (51). These data support our hypothesis that the regulation of FGF21 expression by extracellular ATP would be related to the activation of the PI3K/Akt/mTORC1/ATF4 signaling pathway in muscle. In the current work, exogenous ATP showed a concentration-dependent effect for increasing the FGF21 protein expression in the whole FDB muscle. Interestingly, the maximal mRNA expression was observed with 3 μM ATP, the same concentration that evokes the largest Akt activation (51). The time with exogenous ATP required for Akt activation is 5-7 min, and for mTOR activation 20 min (51), faster than the 30 min required for FGF21 mRNA expression observed in this work. That reinforces the idea of a timeline of molecular events.

The increased levels of mRNA, intracellular protein, and secretion of FGF21 evoked by exogenous ATP were abolished after preincubation with pharmacological blockers of PI3K (LY294002), Akt (Akt VIII) and mTORC1 (Rapamycin). The latter confirms that the PI3K/Akt/mTORC1 pathway is required for the eATP-evoked control of FGF21 expression. That agrees with previously published data that involved these proteins in the regulation of FGF21 expression (4, 36, 64). Previously published data showed the involvement of components of the Akt/mTORC1 pathway in contexts in which the proteins were constitutively activated or blocked with molecular tools and/or transgenic animals. These systems force metabolic pathways and activate cellular stress pathways, which influenced the classification of FGF21 as a myokine only expressed under these stress conditions in skeletal muscle (15, 16, 65). However, our results show the participation of this pathway in a physiological context for skeletal muscle, given that no process has been genetically manipulated to induce a model of metabolic alteration. In the same way, the stimulation carried out with eATP was on muscle cultured in a medium with glucose and aminoacids, so a metabolic stress condition is not generated. Therefore, these results allow us to demonstrate the expression of FGF21 in skeletal muscle under normal exercise conditions, a situation that has remained controversial in the literature. Circulating levels of FGF21 have been reported to increase in response to acute training in mice and healthy humans (23–28). One study shows that acute exercise also increases the FGF21 expression in liver and skeletal muscle in mice and humans (27). However, another study mentions that there are no changes in the expression of muscular FGF21 and that the increase in plasmatic levels after exercise responds only to increased expression of hepatic FGF21 (24). A possible explanation for the latter observation could be that the study of Kim et al. only assessed FGF21 mRNA in skeletal muscle, which may not directly correlate with protein levels. Or on the other hand, they could be evaluating time points in which the kinetics of mRNA increase is not detected. Our approaches on isolated muscle fibers showed that an electrical stimulus that promotes ET-coupling and favors the oxidative fiber phenotype (24) induces both the expression and secretion of FGF21 from skeletal muscle cells.

It has been described that extracellular ATP-mediated signaling induces IP_3_-dependent increases in intracellular Ca^2+^ in skeletal muscle cells (44, 45, 47). In the current work, the participation of Ca^2+^ signals in the regulation of FGF21 was not directly evaluated, which prevents us from suggesting or ruling out the involvement of this second messenger in the pathway proposed for eATP-evoked FGF21 expression. However, it has recently been reported that eATP induces transient intracellular Ca^2+^ signals that induce mTOR activation and protein synthesis, through the modulation of a specific calcium dependent PI3K isoform (66). Accordingly, it is likely that this second messenger could regulate FGF21 expression in a way complementary to the pathway proposed from the current work. Additional studies are required to determine the participation of IP_3_-dependent Ca^2+^ signals in regulating the expression of FGF21 or other myokines in skeletal muscle.

The beneficial effects of FGF21 have been mostly related to its action over adipose tissue and liver (1). Rosales et al. recently demonstrated that FGF21 promotes glucose uptake in skeletal muscle fibers, independent of Akt but dependent on PKC-ζ downstream of PI3K and GLUT4 translocation (38). Those findings, combined with our data that demonstrate FGF21 expression and secretion after electrical stimulation, strongly suggest that FGF21 could be an autocrine/paracrine signaling molecule, secreted during muscle activity to improve glucose uptake for muscle metabolic demands.

Although plasma FGF21 levels have been described as biomarkers of metabolic disorders or mitochondrial myopathies (67–69), its release from muscle has been associated with improvements in metabolic function (42, 70, 71). Consequently, it is complex to assign a favorable or harmful role to the circulating levels of FGF21 “per se” for metabolic health. Apparently, it would depend on the tissue source, type of stimulus, and interaction with other secreted molecules (42). Recombinant FGF21 is unsuitable for clinical use owing to poor pharmacokinetic profiles, short half-life, inactivation in plasma, and instability in solution (reviewed in (70). Therefore, the prescription of specific exercise protocols could be addressed to allow an endogenous increase in plasma FGF21.

In conclusion, the results presented in the current work show for the first time the role of electrical stimulation through extracellular ATP in regulating the expression, synthesis, and secretion of FGF21 through activating the P2YR/PI3K/Akt/mTORC1 pathway in skeletal muscle. These results allow us to identify the extracellular ATP-dependent signaling pathway as a new study target to modulate the production of FGF21 in skeletal muscle and incorporate FGF21 as one of the genes associated with the control of Excitation-Transcription Coupling, acting as a paracrine regulator of muscle plasticity. Determining this ATP-dependent molecular mechanism for regulating FGF21 allows the reopening of the debate on its expression in basal conditions and regulated by physiological stimuli.

## Data availability statement

The raw data supporting the conclusions of this article will be made available by the authors, without undue reservation.

## Ethics statement

The animal study was reviewed and approved by Institutional Animal Care and Use Committee of the Faculty of Dentistry of Universidad de Chile (Certificate N° 061501).

## Author contributions

Conceptualization, MA-C, EJ and SB. Methodology, MA-C, MC, JB-M, CM-J, NH, PL and SB. Formal analysis, MA-C and SB. Funding acquisition, EJ and SB. Data interpretation and discussion, MA-C, MC, PL, EJ and SB. Project administration, NH and SB. Supervision, SB. Writing—original draft preparation, EJ and SB. Writing—review and editing, MA-C, PL, EJ and SB. All authors contributed to the article and approved the submitted version.
